# Use of Online Communities among People with Type 2 Diabetes: A Scoping Review

**DOI:** 10.1007/s11892-024-01538-2

**Published:** 2024-03-08

**Authors:** Arantxa Bujanda-Sainz de Murieta, Nelia Soto-Ruiz, Cristina García-Vivar, Leticia San Martín-Rodríguez, Paula Escalada-Hernández

**Affiliations:** 1 Department of Health Sciences, Public University of Navarre (UPNA), Avda. Barañain S/N, 31008 Pamplona, Navarra Spain; 2 IdiSNA, Navarra Institute for Health Research, Pamplona, Spain

**Keywords:** Diabetes online community; scoping review, Social networking, Type 2 diabetes

## Abstract

**Purpose of Review:**

People with diabetes require continuous self-monitoring and face numerous decisions in their day-to-day lives. Therefore, on many occasions, they need more support than that provided by health professionals. In this context, peer support in online diabetes communities could be a useful tool. The purpose of the review is to describe, analyze and synthesize the available evidence on the use and health out-comes of online communities for people with type 2 diabetes mellitus. A scoping review was conducted in accordance with the Joanna Briggs Institute guidelines. Searches were performed PubMed, Web of Science, CINHAL, Scopus and Cochrane databases.

**Recent Findings:**

From 1821 identified documents, 6 articles were included. These studies explored the characteristics of diabetes online communities and the population features. Besides, the results were classified according to whether they were clinical, psychosocial, or addressed people's experiences with the online community. The analysis underscores their value in facilitating communication, improving diabetes management, and enhancing psychosocial well-being. Future investigations should prioritize longitudinal assessments to elucidate the sustained impact of community engagement and optimize user participation for enhanced patient outcomes.

**Summary:**

The growing relevance of new technologies has led to a significant number of individuals with chronic illnesses seeking peer support. Online health communities have emerged as virtual spaces where individuals with shared health interests interact and form relationships. Within these digital spaces, individuals can engage in peer interaction, observe behaviors, and mutually benefit, potentially leading to improved attitudes toward the disease.

**Supplementary Information:**

The online version contains supplementary material available at 10.1007/s11892-024-01538-2.

## Introduction

Diabetes mellitus (DM) is considered a public health problem of the twenty-first century due to its high incidence and prevalence worldwide, which continues to increase. In 2021, 537 million people had diabetes and it is estimated to reach 643 million people by 2030 [1]. The management of this disease is not straightforward due to the different factors that influence its control. Adherence to drug treatment, dietary control, regular physical exercise, lifestyle changes and continuous glucose measurements, inter alia, are necessary to maintain glucose levels in the appropriate range [2•].

Although people with diabetes have to make many decisions about their disease throughout their lives, they spend no more than 1% of their time in contact with healthcare professionals. Therefore, the self-management of their diabetes is carried out outside the healthcare environment and it is the patients themselves who have to assume control and management of their own treatment [3]. In this sense, both diabetes education and the support provided to them are essential [4]. For this reason, it seems necessary for people with DM to have continuous support beyond the usual check-ups with healthcare professionals.

In this way, peer support can be a promising tool. Peer support is defined as “the giving of assistance and encouragement by an individual considered equal” [5]. People with chronic illnesses often feel more comfortable sharing their experiences and challenges with others with whom they can talk based on their own reality [6]. In addition, sharing experiences can enhance understanding and support learning [7].

In this effort to connect patients with each other, the use of new technologies is becoming increasingly relevant. In fact, according to the Pew Research Center [8], one in four Internet users living with a chronic illness seeks out other people with a similar health condition online. Peer support by using digital tools allows knowledge to be expanded beyond the network of contacts, increasing the possibility of satisfying the needs of the participants [7].

As part of this trend, online health communities have emerged as virtual spaces where a group of people who share a common interest related to health form relationships and interact online [9, 10]. These communities allow users to interact with their peers, observing each other´s behavior and seeking mutual benefit. They also have the potential to improve patients´ attitudes towards their disease, since they could be used as a forum to educate [11].

In the specific field of ​​diabetes, online activity has also grown exponentially. Diabetes discussions began in the 1980s by telephone helpline services, and later, web-based discussion forums and social networks gained prominence. The term “diabetes online communities” (DOC) was first used in 2005 on the blog Six Until Me to refer to online forums and content for people with diabetes and their families [6]. Currently, a DOC is an online community developed to share knowledge and provide support based on the user experience of living with diabetes. It is a tool for people to learn self-care practices from peers, discuss diabetes issues, and connect with others in a similar situation. These communities facilitate open communication and help patients take an active role in their health. In this way, they can be a source of confidence, inspiration, motivation and encouragement [12, 13] ​​. Communities are hosted on “social media platforms”, which are defined as any Internet-based system for the creation, exchange, or distribution of any user-generated content for information, advertising, or any other purpose [14].

Although efforts in the use of technologies have been directed more to people with type 1 diabetes mellitus, possibly due to the characteristics of the population, there are increasingly more patients with type 2 diabetes (T2D) using these tools, so it seems interesting to be able to analyze the existing knowledge to date.

The aim of this study is to describe, analyze and synthesize the available evidence on the use and clinical and psychosocial results of online communities for people with type 2 diabetes mellitus.

## Material and Method

A scoping review was performed, as it was considered the most appropriate methodology to fulfill the purpose of this study. Scoping reviews reach broad objectives and identify areas for further synthesis of results and map the evidence in a currently understudied area. Consequently, it is the ideal format to deepen the use of online communities among people with T2D [15, 16].

The scoping review was established according to the guidelines of the Joanna Briggs Institute [16]. As proposed by Arksey & O'Malley [17], will consist of 5 phases. The results are presented according to the PRISMA- ScR (Preferred reporting items for Systematic reviews and Meta- Analyses extension for Scoping Reviews) [18].

### Phase I: Development of the Research Question

Considering the objective of this review, the research questions are two:

- What are the characteristics of online communities of people with type 2 diabetes?
- What is the evidence for the use of online communities for people with type 2 diabetes?

These questions correspond to PCC-type, a framework that is considered appropriate when developing research questions in scoping reviews [16]. The acronym corresponds to the initials: (P) for Population, (C) for Concept and (C) for Context. The population in this case are people diagnosed with T2D with any type of treatment. And the concept corresponds to online communities. The context is not explicitly stated, as any setting would be eligible [19].

### Phase II: Determination of Inclusion and Exclusion Criteria and Systematic Search

Regarding the inclusion criteria, we included studies that analyzed the use of online diabetes communities in which communication groups could be created (social networks, blogs, online communities, forums…), which had evaluated effects or changes at a clinical or psychosocial level in patients regarding peer support and whose population were people with T2D. The search filtered those articles published from 2012 to February 7, 2024.

Furthermore, we excluded literature reviews, interventions that did not have a digital component, and studies in which the intervention did not have a peer-to-peer interaction, as they did not match our initial definition of online diabetes communities. Studies that did not evaluate results derived from the interaction between peers (evaluation of educational programs, assessment of medical records by health professionals…) were also excluded.

#### Search Strategy

Finally, regarding the search strategy, we used Pubmed, Web of Science, CINHAL, Scopus and Cochrane databases with the following keywords: diabetes mellitus type 2, diabetes type 2, type 2 diabetes, social media, social networking, online social network, patient portal, social medium, web 2.0, patient web portal, patient internet portal, online community, online peer support community, forum, blog, online. The search was limited to those articles that contained the aforementioned terms in their title or abstract. They were combined using the Boolean operators “AND” and “OR”. Annex 1 shows the search strategy in detail. The Zotero application v.6.0.19 (Zotero.org) was used to manage the references.

### Phase III: Review and Selection of Studies

Two reviewers participated in the study selection process. Discrepancies were resolved by consensus or by the intervention of a third reviewer.

**Fig. 1:**
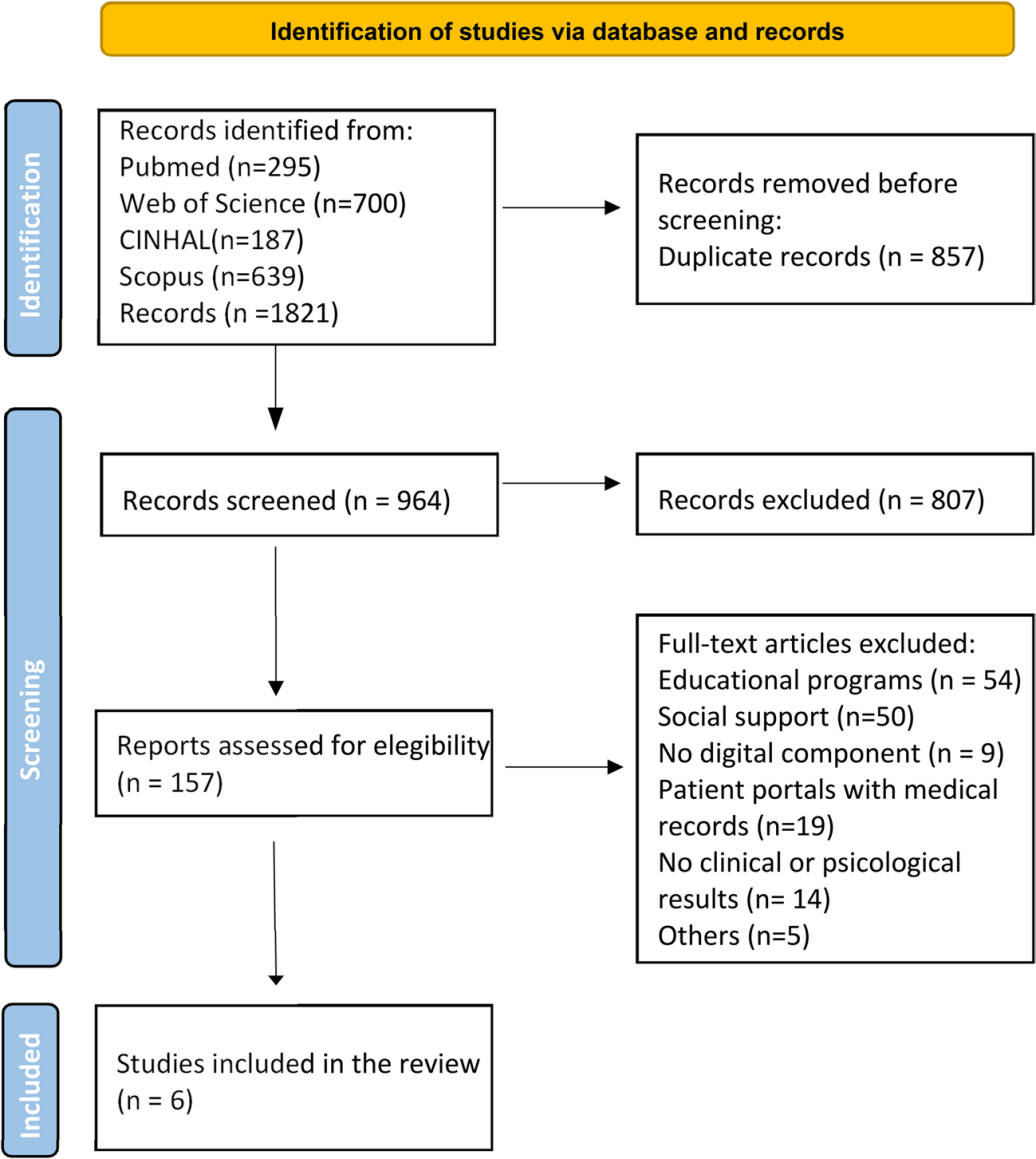
PRISMA 2020 flowchart. Page MJ, McKenzie JE, Bossuyt PM, Boutron I, Hoffmann TC, Mulrow CD, et al. The PRISMA 2020 statement: an updated guideline for reporting systematic reviews. BMJ. 2021;372:n71. 10.1136/bmj.n71

Firstly, duplicate studies were identified and removed. Next, the titles and abstracts of the articles were reviewed, and a second elimination round was performed using the inclusion and exclusion criteria discussed above. Finally, the full texts of the studies were retrieved and the selection was made applying the aforementioned criteria. For those articles included in the analysis, a search of the reference lists was performed to detect relevant studies that were not captured in the first database search. A flow chart detailing the reasons for exclusion of the articles selected for full-text reading is included in Fig. 1 [18].

### Phase IV. Data Extraction

In the data extraction process, a specific table was created according to the needs of the review: name of the first author, year and country of publication, objective, design, sample and inclusion/exclusion criteria, intervention and duration. In addition, the main results were analyzed.

### Phase V. Analysis and Reporting of Results

The aim of the scoping review was to present an overview of the selected studies. For this purpose, a narrative account was conducted. A scoping study does not seek to assess the quality of the evidence and, therefore, cannot determine whether particular studies provide robust or generalizable findings [17].

A description of the general characteristics of the studies included in the review was made, followed by a synthesis of the main characteristics of the online diabetes communities and the results reached in these investigations.

## Results

Five databases were used in the search, through which 1821 results were obtained. Once duplicate articles were removed, 964 studies were screened for relevancy. After reading the title and abstract, 157 were read completely. Of these articles, 151 were excluded for the following reasons: 54 of them were educational programs without peer interaction, 50 dealt with the importance of social support from friends and family and not between patients, 9 had no digital component, 19 evaluated a patient portal where they uploaded their medical records and communicated with healthcare professionals, 14 articles did not yield clinical or psychological outcomes and 5 were removed for other reasons (they did not talk about T2D, were communications of already selected articles, etc.) (Fig. 1). The remaining 6 studies were considered eligible for this review. The main characteristics of the studies are shown in Table 1 [20].

**Table 1:** Characteristics and results of studies of online communities in people with T2D

Author (year) and country	Objective	Design	Sample and inclusion/exclusion criteria	Intervention and duration
Herrero et al. (2021); Spain [21•]	To find connections between participation in diabetes-related online communities and self-reported degree of self-care management and health problems associated with type 1 and type 2 diabetes.	Cross-sectional study	*n* = 307 Inclusion: diabetes, provide consent to participate in the study. Exclusion criteria are not mentioned.	Online surveys of people with DM to see the differences between people who belonged to an online community and those who did not. The interviews were conducted at a certain point, without any follow-up.
Johnson et al. (2014); United States [22]	To evaluate the feasibility and efficacy of participation in a digital virtual environment for diabetes self-managed diabetes education and support (DSMES).	Longitudinal study (pre-middle-post of a single group)	n = 20 Inclusion: between 21 and 75 years old, able to speak English, computer literate, had access to internet connection, reachable by telephone, had no comorbidities or severe diabetes-related complications, and able to travel to the clinic for follow-up appointments. Exclusion criteria are not mentioned.	SLIDES was designed to promote self-management and support based on social cognitive theory. 12 weekly classes were offered and a nurse moderated a weekly support group. Follow-up: 3 and 6 months.
Kim et al. (2022); South Korea [23•]	To research the effectiveness and understand the process of a nurse-led social media intervention for health behavior and glucose control for diabetes self-management.	Mixed methods study (sequential explanatory design) including a randomized control trial.	n = 89 Inclusion: T2D, aged 19 years or older, had poor diabetes control (HbA1c > 7.0%), in possession of a smartphone, and able to read and communicate in Korean. Exclusion: people with cognitive impairment and those who could not use applications on a smartphone by themselves.	In this online community, nurses uploaded diabetes self-managed information and provided interactive support. They were asked to upload their own action plans every Monday. Follow-up: 3 and 6 months.
Verma et al. (2019); India [24]	To assess the use and benefits of social media among people with T2D in India as a well-being mechanism.	Ethnographic study	Observation of 4 online communities and content analysis. Online interviews with 8 participants.	Four communities of people with diabetes previously created on Facebook were included. Online community activities and content analysis were observed. Interviews at a certain point were also conducted.
Litchman et al. (2022); United States [25•]	To assess the feasibility, acceptability and satisfaction of a continuous glucose monitoring intervention together with an online community and report key recommendations to improve the intervention.	Mixed methods (sequential explanatory design)	n = 26 (46 patients initially. Loss of follow-up = 20) Inclusion: age > 20 years, Hispanic, with T2D without insulin and with Internet access and willing to participate. Exclusion: use of continuous glucose monitoring (CGM) in the previous 6 months, illness that prevented participation.	Participants used continuous glucose meters and an existing online T2D community (Beyond Type 2) for 12 weeks. They were encouraged to participate in the community at least 3 times per week. They completed a semi-structured post-intervention interview to document their experiences and provide feedback.
Litchman et al. (2022); United States [26]	To assess participants' experiences with continuous glucose monitoring and the use of online peer support communities.	Qualitative descriptive interpretative study		

Original table created by the authors

The results of the analysis of the studies are presented below, structured into the following sections: characteristics of the DOC, clinical outcomes, psychosocial outcomes and experiences of people with diabetes in using online communities.

Regarding the country of the studies, 50% (*n* = 3) came from the United States, and the remaining three studies were from South Korea, India and Spain.

### Characteristics of Online Diabetes Communities

In this section we will explain the general aspects of the online communities analyzed, how they are dynamized and which members are in charge of moderating them and, finally, how people with T2D evaluate them.

#### General Features

Online diabetes communities can be set up on specific platforms or use available social networks. Related to this issue, both studies by Litchman et al. [25•, 26] and Herrero et al. [21•] used a diabetes-specific online community created on a web domain, while the other authors studied communities created on broader social networks such as Facebook [21•, 24]; Never Cafe [23•] or Second Life [22]. All of them still active.

Once we analyzed in more detail the characteristics of online communities, we found that the studies led by Litchman [25•, 26] used Beyond Type 2 [https://community.beyondtype2.org/), an online community created in 2009 by the non-profit organization Beyond Type 1. This community requires pre-registration for people with T2D to share their stories, connect with each other, and find resources on topics ranging from daily management to mental health management. The community has a group exclusively for Spanish-speaking people, tailored to the culture, customs and traditions. In addition, an email with new posts or news is sent monthly.

Nevertheless, in two of the articles, specific Facebook groups previously created are used [21•, 24]. As it is widely known, Facebook is a social network that requires the creation of a user to access. Facebook groups, which can be public or private, are small groups that allow quick and easy organization of people with similar interests or characteristics, who can share messages, images, and material on the group's wall. Both “Diabetes support” (https://www.facebook.com/groups/MyDiabetes/about) and “Diabetes contact group” (https://www.facebook.com/groups/1169185889761308) were created in 2016, to provide support and information about diabetes in addition to connecting people living with diabetes or their family and friends. The LCHF group (https://www.facebook.com/groups/LCHF4LIFE) originated in 2014 and focuses on learning and discussing the low-carbohydrate diet [24]. All of them are still active.

Never Café (https://cafe.naver.com/) is one of the most popular social networks in South Korea, very similar to the previous one. On this platform, the authors developed the community “Diabetes, my companion” with four main activities: diabetes information, action planning in everyday life, patients´ chat room and questions and answers [23•].

Johnson et al. [22] created SLIDES (Second Life Impacts Diabetes Education & Self-Management), an ad-hoc community within Second Life social network (https://secondlife.com/), a virtual world founded in 2003, in which, unlike the previous ones, you can interact with other people through a personalized avatar and exchange virtual products. The SLIDES community provided self-care education and support based on social cognitive theory according to the user´s characteristics. It contained different resources such as a bookstore with links to buy books and links to web pages. In addition, there were restaurants with nutritional information, a gym with exercise videos, and a community center with a classroom, a slide projector, and a forum that allowed participants to share information and personal experiences. Training classes on diabetes were also offered in this virtual center.

The description of the online communities used by the selected studies is summarized in Table 2.

**Table 2:** Description of the online communities analyzed in the studies

	Online communities
Litchman et al. (2022) [25•]	Beyond Type 2 (https://community.beyondtype2.org/): an established community for people with diabetes created in 2009 by the non-profit organization Beyond Type 1. This community requires pre-registration for people with T2D to share their stories, connect with each other, and find resources on topics ranging from daily management to mental health management. The community has a group exclusively for Spanish-speaking people, tailored to the culture, customs and traditions. In addition, an email with new posts or news is sent monthly.
Litchman et al. (2022) [26]	
Herrero et al. (2021) [21•]	Online communities to which the participants belonged included: Facebook groups, diabetes-related forums, health-related forums, and other online forums.
Verma et al. (2019) [24]	Diabetes support: a Facebook group with approximately 27,000 members Diabetes contact group- a diabetic support group: Facebook group of about 7,000 members. Both were created in 2016, to provide support and information about diabetes in addition to connecting people living with diabetes or their family and friends. LCHF- Low Calorie, High Fat diet: Facebook group where this diet as a solution to diabetes is discussed and other lifestyle-related diseases. Private community: of dubious nature. It is used in the article to see how and in what ways even dubious ideas about medicine can influence people. It was originated in 2014 and focuses on learning and discussing the low-carbohydrate diet.
Kim et al. (2022) [23•]	Diabetes, my companion: Group within the social network “Never Café”, one of the most popular social networks in South Korea. The contents of this page included: information about diabetes, planning of actions in daily life, chat room for patients and questions and answers.
Johnson et al. (2014) [22]	SLIDES- (Second Life Impacts Diabetes Education & Self-Management), a virtual world founded in 2003, in which, unlike the previous ones, you can interact with other people (adults with T2D and healthcare professionals) through a personalized avatar and exchange virtual products. It was designed to provide diabetes self-management training, with weekly classes and other resources.

Original table created by the author

#### Dynamization of Online Diabetes Communities

In online communities, patients support each other. However, sometimes a figure to moderate the group is necessary. The details of the dynamization are shown in Table 3.

**Table 3:** Description of the dynamization of online communities

Online community	Moderated by	Details
Beyond Type 2 diabetes [25•, 26]	Five peer facilitators with diabetes	They were previously trained and experts in the use of CGM and DOC, who encouraged discussions on self-care behaviors. In addition, the community included three weekly intervention-related postings that focused on describing a personal experiment and goal setting; goal recording and problem-solving; and a final goal review.
Facebook groups [21•, 24]	It is not detailed whether there was moderation or not	Observed communities
Diabetes, my companion [23•]	Diabetes nurse educators	Based on social cognitive theory, they provided information on self-management (diet, exercise, glucose monitoring, medications, and other occasional health issues) and provided interactive support.
SLIDES [22]	Specialized nurses, diabetes educators or invited health professionals	These professionals gave 12 weekly classes in the virtual world of the Second platform Life, one hour long, using the American Diabetes Association/American Association of Diabetes Educators self-management training about: healthy eating, staying active, glucose monitoring, medications, problem solving, healthy coping, and risk reduction. In addition, a nurse moderated a weekly support group scheduled in this community.

Original table created by the author

#### Use of Online Communities by People with T2D

Regarding the use of online communities, three of them reported different metrics to measure the degree of use [22, 23•, 25•]. Johnson et al. [22] tracked engagement rates and use. Participants logged in to the community an average of 38 times, with an average of 43 min per session, during the 6 months they participated in the study. 75% of logins occurred within the first three months and the majority within the first month. 75% of items such as food, books, menus, web pages, videos, and pharmacy items were "handled" by 19 of the participants while in the community. Participants interacted with these items a total of 1,180 times.

In order to assess participation, Kim et al. [23•] used the summary statistical report of the platform Never Café. The average number of monthly visits of each person to the platform was 8.53. Eighty-four of participants revisited the site within 12 months. And within the different sections of the website, 69.7% returned to search for information on diabetes, 67% to action planning, 45% to the questions and answers section, and 73.3% to the chat. In addition, all verbal and text communications were continuously recorded for the duration of the study. Regarding communication, the members used their voice most of the time when they were with other people and spent most of the time in the classroom (48.6%) where classes were held twice a week.

Litchman, Ng, et al. [25•] analyzed the engagement rate, defined as 3 or more daily messages per participant. The highest rate was 61.5%, with 13 active participants in the forum. They also received a daily e-mail with a summary of new messages, but it was not possible to check their reading.

In both Litchman studies [25•, 26], although they were encouraged to participate 3 times a week, they did not specify if they complied or not. Herrero et al. [21•] only asked if they were members or not and since when, and did not measure the degree of participation. Verma et al. [24] indicated that they interviewed people who were actively involved in the DOC, but did not define what active participation was indeed.

### Evidence in the Use of Online Diabetes Communities

The following sections describe the main clinical and psychosocial results addressed in the selected studies about online diabetes communities.

#### Clinical Results

In the studies analyzed, different clinical data were collected from patients, both directly related to diabetes and to general health condition, to assess the influence of the use of online communities on them (Table 4).

**Table 4:** Favorable clinical outcomes in results in the use of online communities

Author; year	no	HbA1c	Fasting blood glucose	TIR	BP	BMI Weight	TG	Total cholesterol
Johnson et al. 2014 [22] Single group pre-post design	twenty	○			○	○		
Kim et al. 2022 [23•] Intervention group vs. Control group	89	●	●		○		●	○
Litchman et al. 2022 [25•] Litchman et al. 2022 [26] Single group pre-post design	24	○		○				

Original table created by the author

● Statistically significant changes

○ No statistically significant changes

*HbA1c* glycosylated hemoglobin, *TIR* Time in Range, *BP* Blood Pressure, *GT* Triglycerides, *CGM* continuous glucose monitors

In order to analyze the improvement in glycemic control, changes in glycosylated hemoglobin (HbA1c), fasting blood glucose, and time in range were measured. Johnson et al. [22] observed a decrease in HbA1c of 0.59%, although it was not significant, while in the study by Kim et al. [23•] the intervention group presented a significantly lower level than the control group, for non-insulin users, at 3 (6.38; SD (Standard Deviation) = 0.34 vs. 7.25; SD = 0.24, *p* = 0.040) and 6 months (6.31; SD = 0.37 vs. 7.28; SD = 0.26, *p* = 0.036). With the same objective, they recorded fasting blood glucose, in which there were significant improvements in insulin-dependent people who participated in the intervention group at 6 months (135.80; SD = 12.37 vs. 175.82; SD = 15.34, *p* = 0.049). Also Litchman, Ng, et al. [25•] demonstrated that nine participants (37.5%) had a decrease of more than 0.5% HbA1c at 12 weeks, five (20.8%) had an increase of more than 0.5%, and 10 (41, 6%) had no significant changes. They used the time in range (between 70 and 180 mg/dl) in the same way, in which there were no statistically significant improvements either. However, there was an increase in glycemic variability (Z =  − 2.172, *p* = 0.03).

Kim et al. [23•] found significant differences in the reduction of triglycerides (206.85, SD = 38.26 vs. 387.50, SD = 56.19, *p* = 0.013). However, there were no differences in the other measured outcomes such as blood pressure, weight, Body Mass Index or total cholesterol.

Nevertheless, Herrero et al. [21•] reported a greater number of complications derived from diabetes in people who used the DOC. There were differences between groups M = 1.95, SD = 1.28, for people with T2D not belonging to DOC and M = 4.77, SD = 0.72, for DOC users.

#### Psychosocial outcomes

Psychosocial data related to participants´ perceptions such as self-efficacy, self-care, social support, perceived presence and co-presence, quality of life, and knowledge about diabetes were collected in all studies (Table 5).

**Table 5:**
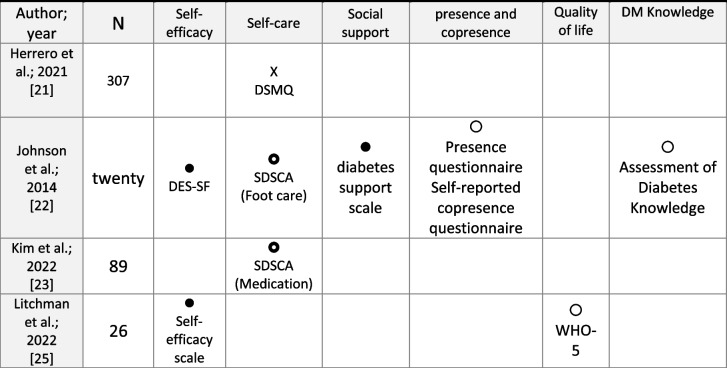
Psychosocial results

Original table created by the author

Statistically significant changes (improvement of parameters in DOC users)

No statistically significant changes

Statistically significant changes (improvement of parameters in DOC users) in some areas of the scale

X Statistically significant changes (worsening of parameters in DOC users)

*DSMQ* Diabetes Self-Management Questionnaire, *DES-SF* Diabetes Empowerment Scale-Short Form, *SDSCA* Diabetes Self-care Activities, *WHO-5* 5-item World Health Organization Well-Being Index

Regarding self-efficacy, Johnson et al. [22] used the Diabetes Empowerment Scale -Short Form (DES-SF). In a range from 1 to 5, at baseline of the study it was 3.89 points on average (SD = 0.81); however, it improved at 3 months (4.45, SD = 0.67, *p* = 0.036) and at 6 months (4.64, SD = 0.39, *p* = 0.02). While Litchman, Ng, et al. [25•] used the Self-efficacy Scale, whose score significantly improved after 12 weeks of community participation (68.6, SD = 18.3 vs. 82.7, SD = 14.5; *p* < 0.01).

Self-care was evaluated in three studies. Herrero et al. [21•] were based on the DSMQ (Diabetes Self -Management Questionnaire) by four subscales: glucose control, dietary control, physical activity, and use of the healthcare system; as well as a global score of all of them. People with T2D not belonging to online communities (32% of the sample) had a significantly higher score for diabetes self-management in all subscales: glucose control (4.34 vs. 2.51; *p* = 0.000), diet control (4.03 vs. 2.17; *p* = 0.000), healthcare (4.35 vs. 2.58; *p* = 0.000), physical activity (3.91 vs. 1.89; *p* = 0.000), diabetes care (4.05 vs. 2.90; *p* = 0.000); being 0 the minimum score and 10 being the maximum score. Both Johnson et al. [22] and Kim et al. [23•] assessed self-care using the Diabetes Self-Care Activities Scale (SDSCA), made up of 17 items that measure the number of days per week on which the indicated behavior is performed. It presents six subscales on a scale from 0 to 7: general diet, specific diet, physical activity and exercise, blood sugar testing, foot care, and medication. In the first of them, participants only showed a statistically significant improvement in the number of days per week spent to foot care. At baseline 3.68 days on average (SD = 2.08) and at 6 months 6.17 days (SD = 2.08) (*p* = 0.03). In contrast, in the study by Kim [23•] the only significant difference was in the improvement in medication behavior for insulin users in the intervention group at 3 and 6 months. (6.79, SD = 0.34 vs. 5.03, SD = 0.49, *p* = 0.004; 6.37, SD = 0.52 vs. 4.68, SD = 0.65, *p* = 0.047; respectively) [23•].

## Discussion

The aim of this study was to describe, analyze and synthesize the available evidence on the use and health outcomes of online communities for people with T2D. A scoping review was performed to this end, in which six primary studies were identified that addressed the evidence on the use of online communities and its influence on health outcomes for people with T2D between 2012 and 2023. These findings showed a paucity of research regarding these types of resources and interventions in T2D.

In recent years, there has been a growing interest in this type of intervention, specifically for people with diabetes. Thus, our review complements the synthesis of evidence already offered by previous reviews in this area. The systematic review by Elnaggar et al. [27] seeks to describe the use of peer-to-peer social networks to manage diabetes and cardiovascular disease, in addition to evaluating clinical outcomes, behavioral outcomes, quality of life, and patient self-efficacy as a result of the use of peer-to-peer social networks until 2019. However, they do not analyze any intervention specifically in T2D, as is the focus of the present review. Litchman, Walker et al. [3] performed a scoping review focusing on variations in study design, platform and user characteristics of DOC, and potential or actual benefits and consequences. The search was carried out until 2018, while the communities have been growing rapidly and steadily. In order to update their previous research, they used a rapid review in 2021 [28•]. Although they aim to highlight the clinical impact of participation in DOCs and provide guidance to healthcare professionals in navigating and recommending these communities, they only analyze one of the selected studies in this review, as four of them are conducted after 2021 and do not study the interventions of Johnson et al. [22] and Verma et al. [24].

In this study, the concept of online communities was understood as virtual places where peer support is given. Therefore, other types of communities that were based on communication with health professionals or those focused exclusively on diabetes education were not taken into account.

Lewinski et al. [29], in their integrative review, concluded that an ideal community includes a large and diverse sample, a moderator that promotes interaction, synchronous and asynchronous communication, self-management information, and a dynamic learning environment. Despite the fact that the evaluated communities were heterogeneous, they meet these characteristics, except for the role of moderator which does not appear in the study by Verma et al. [24] nor in the study by Herrero et al. [21•]. On the contrary, all communities were included in broader social networks or platforms with an established structure.

Regarding the recruitment of people who participated in the studies, a convenience sampling was carried out in all of them, so it is important to take this limitation into account. The average age of the samples, which varies from 49 to 55 years, coincides with the profile of people with T2D, since the highest prevalence occurs for people between 50 and 59 years [1]. However, the range age of the people who participated in the studies is very wide (from 19 to 81 years) and the results divided into age intervals are not specified. This aspect could be relevant to the results, since older people have to make a greater effort to adapt to the use of information and communication technologies, which have considerably increased since the year 2000 [30]. Although social networks are becoming increasingly important in mitigating loneliness for elderly people, further research is needed to adapt existing technology and incorporate emerging technology into care therapies. In fact, these tools could transform the way people with diabetes are supported to improve their self-management [31].

This review analyzes clinical results, psychosocial outcomes and/or experiences with online diabetes communities (use, satisfaction, difficulties…). Regarding the clinical repercussion, it has been verified that people with diabetes have from 2 to 4 times increased risk of stroke and death from heart disease [32]. Elevated HbA1c level has been identified as a significant risk factor for cardiovascular disease and stroke. In fact, a 1% increase in HbA1c concentration was associated with an increase in mortality of about 30–40%. Whereas reducing the HbA1c level by 0.2% could reduce mortality by 10% [33]. In the analyzed studies that evaluated HbA1c, an improvement greater than 0.5% was observed [2•, 22, 26]. However, the improvement is only significant in the study by Kim [34], possibly due to the sample size. It is also noteworthy that, in a 12-week intervention, such as that of Litchman, Ng, et al. [25•], it is not easy to see changes in glycosylated hemoglobin, which measures the average level of glucose over three months. Similarly, in the study by Kim et al. [23•], there is a significant drop in triglyceride levels, also associated with cardiovascular risk. These clinical findings correspond with the results of other online communities for people with type 1 diabetes mellitus, where they demonstrate improvements in HbA1c [35–37] and cholesterol levels [38].

In the same way, psychosocial improvements in a chronic disease such as diabetes are of the utmost importance. Although the concepts of self-care and self-management have different nuances, in the analyzed articles they are used interchangeably [39]. In the studies by Johnson et al. [22] and Kim et al. [23•] they used the SDSCA scale, indicating significant improvements in the subscales of foot care and medication taking, respectively. However, in the intervention by Herrero et al. [21•] the level of self-care, measured by the DSMQ questionnaire, was lower in those people who used the DOCs.

Regarding self-efficacy, understood as an individual's confidence in their ability to carry out specific self-management behaviors, it significantly increased in all the studies in which it was evaluated. Johnson et al. [22] used the DES-SF questionnaire, while Litchman, Ng, et al. [25•] do not specify the scale. According to Bandura's self-efficacy theory [40], people who are confident in their abilities are more consistent in maintaining their daily tasks. Similarly, Karimy et al. [41] indicated that self-efficacy is the most important predictor of self-care behaviors in patients with diabetes. Social support also facilitates healthy behaviors and has a direct connection to self-care behaviors of people with diabetes [42]. However, further research is needed to recognize whether these results are a cause or a consequence of participation in DOC. These results are consistent with other support groups in other diseases, such as cancer or mental health, where positive but inconclusive results are shown for the time being [43–45].

Other reviews carried out on online communities in people with T2D, discussed above, also conclude that results are positive and negative consequences are few, but further research should be carried out on the subject, since its use is incipient [3, 29, 30].

Furthermore, although the aim of this review is not to perform a quality analysis, the results should be interpreted with caution, since there are certain limitations in the analyzed studies, such as the participants recruitment, the sample size or the use of self-reported scales.

The present study is not without limitations. One of them is the heterogeneity in the diabetes online communities, making it difficult to draw conclusive conclusions. In addition, the review is limited to scientific articles, being aware that there will be many online communities in which results can be reported by surveys, but no evidence is recorded. Due to the different perceptions about what an online community is, it could be that we have missed some specific virtual place where support is given among patients.

## Conclusions

The analysis of online communities for individuals with type 2 diabetes mellitus (T2D) reveals a variety of features and outcomes across the studies reviewed. Despite the diversity, these communities consistently offer a valuable space for synchronous and asynchronous communication among patients, contributing to improved diabetes management and psychosocial well-being.

Psychosocial outcomes also demonstrated the beneficial impact of online communities on participants' self-efficacy, self-care behaviors, and social support networks. Moreover, the sense of community and support provided by peers and moderators contributed to enhanced well-being and quality of life for participants. Engagement metrics indicated active participation among users, with frequent logins and interactions observed in various online communities. However, improvements in glycemic control were not consistently significant across all studies.

While the findings are promising, it is essential to acknowledge the limitations of the existing research, including small sample sizes and methodological variations. Therefore, future studies should aim to address these limitations and conduct more comprehensive assessments of the effectiveness of online peer support interventions for individuals with T2D. Additionally, exploring the long-term effects of community engagement and identifying strategies to optimize user participation are crucial areas for further investigation.

Given the prevalence of T2D and the proliferation of technology and its influence on social interaction, further investigation into online peer support interventions targeting people with this disease is needed.

Future research should focus on analyzing patient outcomes based on participation in online communities and conduct correlational studies based on this.

### Supplementary Information

Below is the link to the electronic supplementary material.

Supplementary file1 (DOCX 14 KB)
