# Supplementary material for: Use of Online Communities among People with Type 2 Diabetes: A Scoping Review
Source: Curr Diab Rep. 2024 Mar 8;24(5):96–107. doi: 10.1007/s11892-024-01538-2 (PMC11043193; doi:10.1007/s11892-024-01538-2)
Supplement: Supplementary file 1 — Supplementary file1 (DOCX 14 KB) [file 11892_2024_1538_MOESM1_ESM.docx]

**Annex 1**: Search strategy

01/01/2012 - 07/02/2024

| **Pubmed** | **Results** |
| --- | --- |
| ("Diabetes Mellitus, Type 2"[Title/Abstract] OR "Diabetes, Type 2"[Title/Abstract] OR "Type 2 Diabetes"[Title/Abstract] OR "Diabetes Mellitus, Type 2" [Mesh]) AND ("social media"[Mesh] OR "social networking"[Mesh] OR "online social networking"[Mesh] OR "patient portals"[Mesh] OR "social media"[Title/Abstract] OR "social medium"[Title/Abstract] OR "Web 2.0"[Title/Abstract] OR "social networking"[Title/Abstract] OR "online social networking"[Title/Abstract] OR "social media"[Title/Abstract] OR "patient portal"[Title/Abstract] OR "patient web portal"[Title/Abstract] OR "patient internet portal"[Title/Abstract] OR "online community"[Title/Abstract] OR "forum"[Title/Abstract] OR "blog"[Title/Abstract] OR "online social network"[Title/Abstract] OR “online peer support community") | 295 |
| **Web of science** | **Results** |
| TS= (("Diabetes Mellitus, Type 2” OR "Diabetes, Type 2" OR "Type 2 Diabetes") AND ("social media" OR "social network*" OR "online social network*" OR "social medium" OR "Web 2.0" OR "patient portal" OR "patient web portal" OR "patient internet portal" OR "online community" OR "digital platform" OR "forum" OR "blog" OR "online social network" OR “online peer support community")) | 700 |
| **CINHAL** | **Results** |
| AB("Diabetes Mellitus, Type 2” OR "Diabetes, Type 2" OR "Type 2 Diabetes") AND ("social media" OR "social network*" OR "online social network*" OR "social medium" OR "Web 2.0" OR "patient portal" OR "patient web portal" OR "patient internet portal" OR "online community" OR "digital platform" OR "forum" OR "blog" OR "online social network" OR “online peer support community") | 187 |
| **Scopus** | **Results** |
| TITLE-ABS-KEY (("Diabetes Mellitus, Type 2” OR "Diabetes, Type 2" OR "Type 2 Diabetes") AND ("social media" OR "social network*" OR "online social network*" OR "social medium" OR "Web 2.0" OR "patient portal" OR "patient web portal" OR "patient internet portal" OR "online community" OR "digital platform" OR "forum" OR "blog" OR "online social network" OR “online peer support community")) | 639 |
